# Impaired mnemonic pattern separation associated with PTSD symptoms paradoxically improves with regular cannabis use

**DOI:** 10.1038/s44184-025-00126-w

**Published:** 2025-04-24

**Authors:** Jacob Ross, Bruna Cuccurazzu, Dylan Delmar, Christian Cortez, Giovanni Castillo, Dean T. Acheson, Dewleen G. Baker, Victoria B. Risbrough, Daniel M. Stout

**Affiliations:** 1 Department of Biological Sciences, University of California San Diego, San Diego, CA USA; 2 Research Service, San Diego ORD VISN 22, VA San Diego Healthcare System, San Diego, CA USA; 3 Department of Psychiatry, University of California San Diego, San Diego, CA USA; 4 Center of Excellence for Stress and Mental Health, VA San Diego Healthcare System, San Diego, CA USA

**Keywords:** Psychology, Diseases, Signs and symptoms

## Abstract

Posttraumatic stress disorder (PTSD) is associated with poor hippocampal function and disrupted pattern recognition. Cannabis use is highly prevalent in individuals with PTSD, yet the impact on these cognitive functions is poorly understood. Participants (*n* = 111) with a range of PTSD symptoms with and without regular cannabis use completed the mnemonic similarity task. We hypothesized that regular use would be associated with alterations in pattern separation ability in individuals with PTSD symptoms. High PTSD symptoms were associated with reduced pattern separation performance in minimal users. Regular users with high PTSD symptoms showed greater pattern separation, but reduced pattern separation with low PTSD symptoms. These results suggest that regular cannabis use may disrupt pattern separation and similar hippocampal-dependent processes, while it may improve pattern separation in individuals with high PTSD symptoms. These cross-sectional results require longitudinal follow-up studies to evaluate the causal effects of regular cannabis use on cognitive function in PTSD.

## Introduction

PTSD is often characterized as a disorder of ‘fear memory’ due to the chronic and persistent symptoms of intrusive trauma-related memories that disrupt functioning long-after the trauma occurred^1,2^. The impact of PTSD on memory, particularly the contextual modulation of fear memory, has become a focal point of investigation^3–5^. Contextual processing is using internal or external environmental cues to optimize the most appropriate behavioral response^6–9^. One integral component of contextual processing is pattern separation, which is the ability to distinguish between similar experiences and form distinct mnemonic representations^10,11^. By forming distinct memories and retrieving them accurately, pattern separation supports adaptive behavior in various contexts and enhances the ability to navigate and interact with the environment^12,13^. However, dysfunction in the ability to properly distinguish between similar but distinct cues might contribute to the persistence of traumatic memories and overgeneralization of fear responses to non-threatening stimuli that share similarities with stimuli associated with the traumatic event^3,5,14,15^.

Both preclinical and clinical studies have highlighted the role of bidirectional hippocampal-prefrontal cortex interactions in both retrieving past contextual associations and new contextual learning^16–19^. In particular, the hippocampus is implicated in mnemonic pattern separation^10,13,20,21^. PTSD is associated with structural and functional hippocampal dysfunction^22–24^, and has been implicated as both a pre-trauma risk factor for PTSD as well as a potential consequence of PTSD symptoms^25–28^. Collectively, these investigations indicate that contextual processes supported by the hippocampus, including pattern separation, are altered in individuals with PTSD^29^. Consistent with this hypothesis, compared to healthy control participants, treatment-seeking patients with PTSD demonstrate decreased performance in assessments of mnemonic pattern separation ability^30^.

In addition to PTSD, preclinical and clinical studies indicate that heavy cannabis use is also associated with memory impairment^31–36^. The hippocampus has a high concentration of cannabinoid receptors^37–39^, suggesting long-term use of cannabis may alter functions subserved by the hippocampus^40^. Results from numerous cross-sectional studies support an association between heavy cannabis use and reduced hippocampal volumes in both psychiatrically healthy individuals and those with various psychopathology including schizophrenia^41–44^. Some longitudinal studies suggest that long-term use of cannabis is associated with reductions in performance on numerous cognitive tasks and lower hippocampal volume compared to non-users^45,46^. However, some longitudinal investigations and meta-analyses examining both hippocampal volume and cognitive performance differences between cannabis users and non-users have shown mixed results^47–54^.

With the increase in the availability of cannabis, there is a great need to understand the impact of regular cannabis use on contextual learning mechanisms in individuals with PTSD. PTSD and problematic cannabis use are highly comorbid. Among a sample of returning US OEF/OIF Veterans diagnosed with Cannabis Use Disorder (CUD), more than 70% also had comorbid PTSD^55^. Results from the 2019-2020 National Health and Resilience in Veterans Study (NHRVS) showed that among Veterans with a current PTSD diagnosis, 28.9% self-reported cannabis use in the previous 6 months, compared to 11.9% of Veterans without PTSD^56^. Many individuals report using cannabis to manage symptoms, particularly sleep and anxiety^57,58^. It is unknown what daily/regular use of cannabis might do to hippocampal functions such as context-dependent pattern separation, a critical process governing contextual modulation of trauma memory and fear generalization^3,5^. Here we tested the hypothesis that regular cannabis use would be associated with significant disruptions in pattern separation memory processes in individuals with elevated PTSD symptoms. To test this hypothesis, we examined performance on the Mnemonic Similarity Task (MST), a widely used test reflective of hippocampal function^59^, in individuals reporting either minimal or regular cannabis use in a sample of trauma-exposed individuals with varying levels of PTSD symptoms.

## Methods

### Study population

Data for the current study were collected from two independent samples. For the first sample, participants (*n* = 90; *M*_age_ = 31.5, SD = 9.51; 34.44% female) with and without PTSD and with minimal or regular cannabis use were recruited to participate in a 2-day fear learning study (data not reported here). We supplemented this sample with a second cohort of participants (*n* = 33; *M*_age_ = 34.76, SD = 5.67; 100% male) from the Marine Resilience Study (MRS)^60^ who matched the same inclusion criteria as the first sample and who also completed the MST. Study protocols and procedures were approved by the Institutional Review Board at the University of California, San Diego, and complied with ethical standards involving human research participants. All participants provided written informed consent prior to participation.

Both cohorts were instructed to refrain from substance use before the testing day. Cannabis use was tracked by urine test in the first cohort with the minimal user group required to have a negative urine test at intake and both groups required to have a negative saliva test (Dräger Drug Test 5000, Houston TX) on the day of testing. Cannabis use assessment in the smaller MRS cohort was limited to self-report due to regulatory limitations. The combined study group consisted of 123 participants, all of whom were free from family or personal history of bipolar or psychotic disorders and antipsychotic medication use. Cannabis use group criteria were based on prior work^61^, with Regular Cannabis Users (*n* = 49 defined as using cannabis at least 3 times/week for >3 months or 2 times/week for > 6 months, and Minimal Users (*n* = 74) as abstaining from cannabis use for > 2 months. See Table 1 for details of cannabis use in the Regular User group.

**Table 1:** Study Sample Demographics

	Minimal Cannabis Users (*n* = 69)	Regular Cannabis Users (*n* = 42)
Age (SD)*	34.16 (8.82)	29.59 (7.94)
**Sex**	26.09% women	30.95% women
**Race**		
Black/African American	8.70%	23.81%
American Indian/Alaskan Native	0%	2.38%
Asian	14.49%	4.76%
Native Hawaiian/Pacific Islander	1.45%	4.76%
White	68.67%	35.71%
Other/Not Reported	8.70%	28.57%
**Ethnicity**		
Not Hispanic or Latino	84.06%	61.90%
Cuban	0%	0%
Mexican	8.70%	16.67%
Puerto Rican	0%	4.76%
South/Central American	2.90%	7.14%
Other Spanish Culture	4.35%	9.52%
**PCL-5 Total Score (SD)**	26.7 (22.3)	33.48 (21.93)
Re-experiencing cluster	7.06 (6.10)	8.67 (6.16)
Avoidance cluster	2.97 (2.73)	3.81 (2.70)
Negative Cognition & Mood cluster*	7.54 (7.65)	11.02 (8.07)
Hyperarousal cluster	8.51 (7.19)	9.88 (6.51)
BDI-II Total Score (SD)*	16.71 (19.6)	24.64 (18.78)
**Cannabis Use (SD; range)**	–	
Grams used/day	–	1.18 (1.11; 0.015–5)
THC content	–	31.2% (16%; 15–90%)
Days use/week	–	4.86 (2.09; 1–7)
Years used at this rate	–	4.28 (5.44; 1–31)

Demographics and characteristics of sample used in the primary analyses. *PCL-5* Posttraumatic Stress Disorder Checklist for the DSM-5. *BDI-II* Beck Depression Inventory, 2nd edition.

*SD* standard deviation.*p < 0.05.

From the combined study group, one outlier from the minimal cannabis user group was removed (>Q3 + 1.5*interquartile range), one individual was removed due to incomplete self-report cannabis use information, and one individual was removed due to missing self-reported PTSD symptoms. Additionally, 9 participants were excluded for having a negative lure discrimination index on the MST, which past work has suggested is indicative of a high response bias and inadequate encoding or retrieval of the initial target object^62^, leaving a total of 111 participants remaining for the final analysis (Minimal Users = 69; Regular Users = 42). The combined sample-size used in the current study provides 80% power to detect a medium-to-large effect size (*Cohens f* = 0.27) for main effects and interactions between two groups and one continuous variable (*α*<.05, *F* tests; G*Power version 3.1.9.6).

### PTSD Checklist for DSM-5 (PCL-5)

The PTSD Checklist for DSM-5 was used to assay PTSD symptom severity. The PCL-5 is a 20-item self-report measure assessing past-month PTSD symptoms (Range: 0–80, >33 indicates probable PTSD diagnosis)^63^.

### Beck Depression Inventory-II (BDI-II)

The BDI-II is a 21-item questionnaire measuring depressive symptoms over the past two weeks (Range: 0–63)^64^.

### Customary Drinking and Drug Use Record (CDDR) – Cannabis Domain

Lifetime and 3-month cannabis use was measured using the Customary Drinking and Drug Use Record (CDDR), which assesses lifetime use, type, and route of cannabis products used, in addition to assessing abuse and dependence criteria, withdrawal effects, and substance-related difficulties^65^.

### Mnemonic Similarity Task (MST)

Participants completed the mnemonic similarity task^59,62,66^. The task consisted of two phases, an incidental learning phase and a surprise recognition test. In the learning phase, participants viewed 192 images (2 s; 0.5 s inter-stimulus interval) on a computer screen. Participants had to respond to each image as an indoor or outdoor object. Immediately following the learning phase, participants completed the recognition test (192 trials). Participants had to respond to each item as “old”, “new”, or “similar.” One third of the trials consisted of images that appeared during the incidental learning phase (targets), one third of the images were new images not presented previously (foils), and one third of the images were similar but not identical to the images that appeared during the incidental learning phase (lures). The primary variable of interest was the lure discrimination index (LDI): [*p*(“similar” |lure) – *p*(“similar”|foil)], which we use as the measure of mnemonic pattern separation performance - the ability to successfully separate similar images from previously viewed images^59,66,67^.

### Analytic approach

Because we measured MST performance from two different studies prior to combining the data for the current manuscript, we sought to reduce the between sample cohort variability. First, we *Z*-scored the MST LDI metric within the sample cohort. Second, we added a sample cohort as a covariate of non-interest. Primary hypothesis testing was conducted by computing a PTSD × Cannabis Use general linear model (GLM). PCL-5 total scores were added as a continuous predictor and Cannabis Use as a categorical variable on the MST LDI (*Z*-Scored). Analyses were performed in Jamovi (R Core Team, 2021; The Jamovi Project, 2022) using GAMLj: General Analyses for the Linear Model in Jamovi (Gallucci, 2019).

## Results

### Participant characteristics

Demographic and participant characteristics are reported in Table 1. When comparing regular vs. minimal users, regular cannabis users did not statistically differ on PCL-5 (*p* = 0.12) but had higher BDI-II total scores (*p* = 0.049; mean difference = 7.48, SE_difference_ = 3.76) and had higher PCL-5 Negative Cognition and Mood symptom cluster scores (*p* = 0.024; mean difference=3.49, SE_difference_ = 1.53). Self-reported PCL-5 and BDI-II were highly correlated (*r* = .80, *p* = 2.2 × 10^-16^). Regular cannabis users were younger than minimal cannabis users (*p* = 0.007; mean difference=4.49 years, SE_difference_ = 1.64). In the regular cannabis use group, participants reported using cannabis 4.86 days/week (SD = 2.09), 1.18 grams (SD = 1.11) per day, and using at this rate for 4.28 (SD = 5.44) years and cannabis use patterns did not relate to PTSD symptoms (grams per day: *r* = −0.04, *p* = 0.80, days/week: *r* = 0.04, *p* = 0.81, years of use at this rate: *r* = 0.12, *p* = 0.45) or to depression symptoms (*rs* < ± 0.02, *ps* > .90).

### Cannabis Use Moderates the Effect of PTSD Symptoms on Mnemonic Pattern Separation

**Fig. 1:**
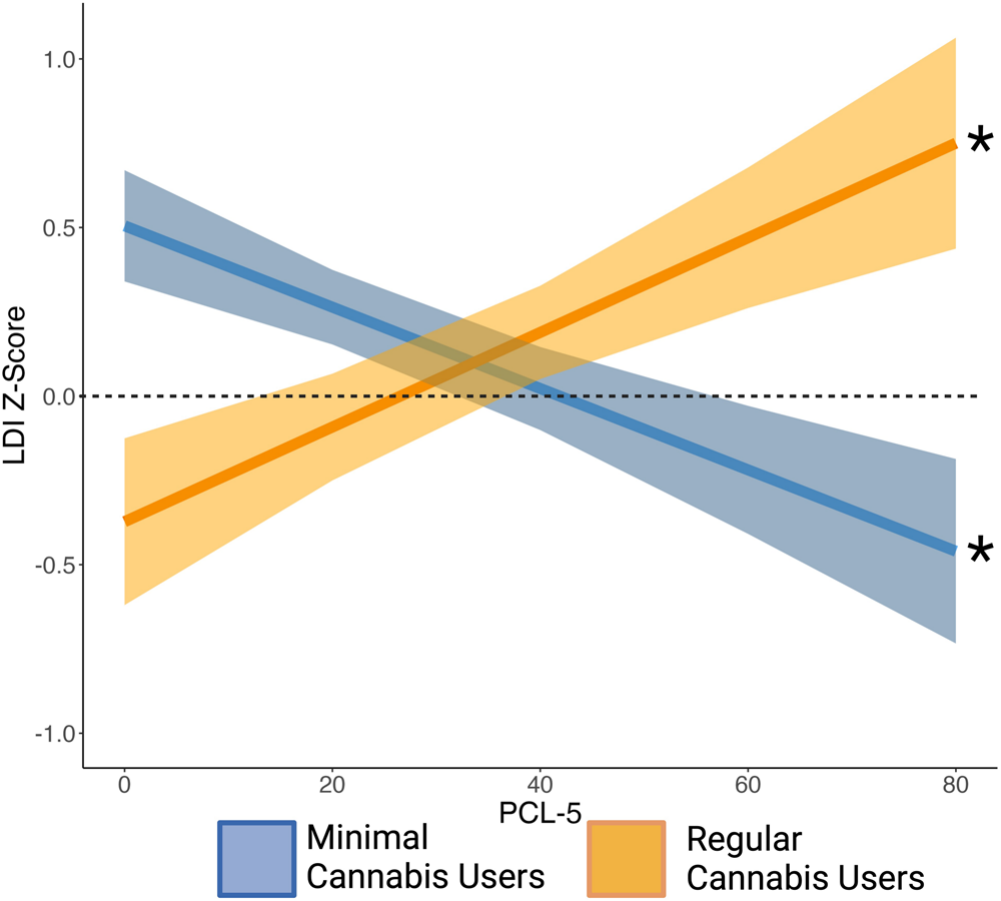
Mnemonic pattern separation performance as a function of PTSD symptom severity in individuals who minimally use cannabis and in individuals with regular cannabis use. LDI Z-Score = Lure discrimination index, Z-scored; which measures mnemonic pattern separation performance. LDI was Z-scored within sample cohort (see Method). PCL-5 = PTSD Symptom Checklist for DSM-5. * Simple slope effects: *p* < .05. Ribbon reflects standard error of the mean. See online article for the color version of this figure. Figure was created using R (*ggplot2*) and BioRender.com.

Results from the GLM indicated that the effects of PTSD symptom levels depended on Cannabis use (See Fig. 1, PTSD × Cannabis Use, *F*(1,106) = 12.38, *p* < 0.001, ${\eta }_{p}^{2}=.10$; Main effect of PTSD *F*(1,106) = 0.002, *p* = 0.97, ${\eta }_{p}^{2} < .001$, Main effect of Cannabis use, *F*(1,106) = 0.26, *p* = 0.61, ${\eta }_{p}^{2}=.004$). The interaction remained significant after controlling for age (*p* < 0.001, ${\eta }_{p}^{2}=.11$) and depressive symptoms (*p* < 0.001, ${\eta }_{p}^{2}=.12$). Simple effects analysis indicated that within the Minimal Cannabis Use group, increasing levels of PCL-5 severity was associated with lower mnemonic pattern separation performance, *β* = −0.341, *t* (106) = −2.91, *p* = 0.004. In contrast, in the Regular Cannabis Use group, increasing levels of PCL-5 severity was associated with greater mnemonic pattern separation performance, *β* = 0.334, *t* (106) = 2.20, *p* = 0.030. These patterns of results were not driven by adding the smaller MRS cohort, the results remained significant when only analyzing the larger subsample recruited for this study (*n* = 79; see Supplementary Table 1).

### Cannabis Use Effects Across Different PTSD Symptom Types and Depression

**Fig. 2:**
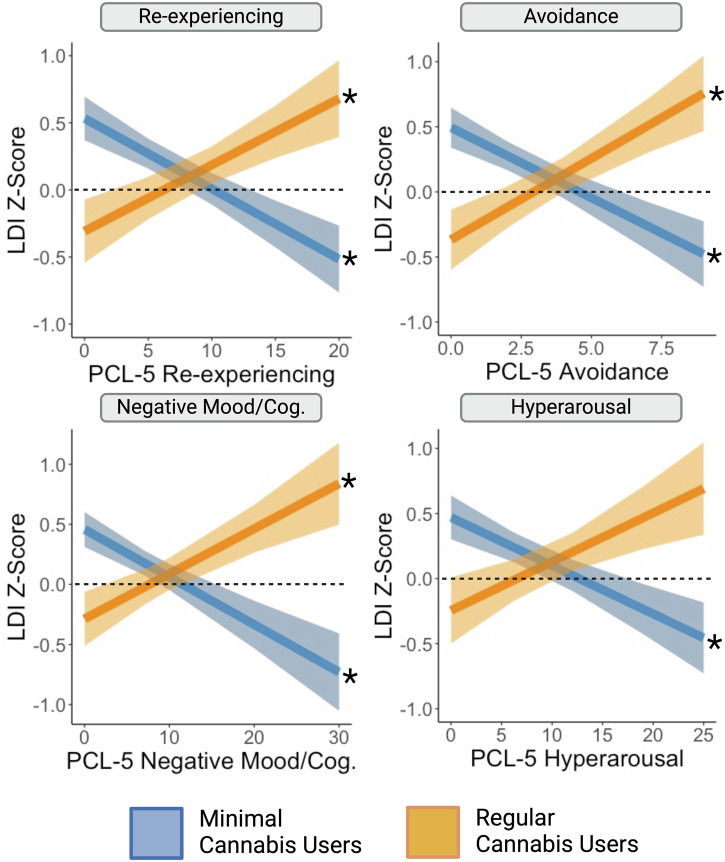
Mnemonic pattern separation performance as a function of PTSD sub-cluster symptom severity in individuals who minimally use cannabis and in individuals with regular cannabis use. LDI Z-Score = Lure discrimination index, Z-scored; which measures mnemonic pattern separation performance. LDI was Z-scored within sample cohort (see Method). PCL-5 = PTSD Symptom Checklist for DSM-5. * Simple slope effects: *p* < .05. Ribbon reflects standard error of the mean. See online article for the color version of this figure. Figure was created using R (*ggplot2*) and BioRender.com.

Next, we further examined the symptom specificity of the findings. First, we computed separate models using each of the PCL-5 symptom subclusters. The effect of increasing mnemonic pattern separation performance with increasing symptom severity in the regular cannabis use group was significant for the re-experiencing, avoidance, and negative cognition and mood PCL-5 subclusters (*p*s < .05; Supplementary Tables 2 through 5; Fig. 2). For the minimal cannabis use group, the effect of decreasing pattern separation performance was significant in all four PCL-5 symptom subclusters *(ps* < *.05)*. Second, when depression symptoms were added as a covariate, the improved pattern separation performance with elevated PTSD symptoms in the Regular Cannabis Use group was no longer significant, *β*=-0.003, *t*(104) = -0.01, *p* = .99; whereas the decreasing performance in the Minimal Cannabis Use group remained significant (*p* < *.05*).

### Regular Cannabis Use Associated with Poor Pattern Separation Ability

**Fig. 3:**
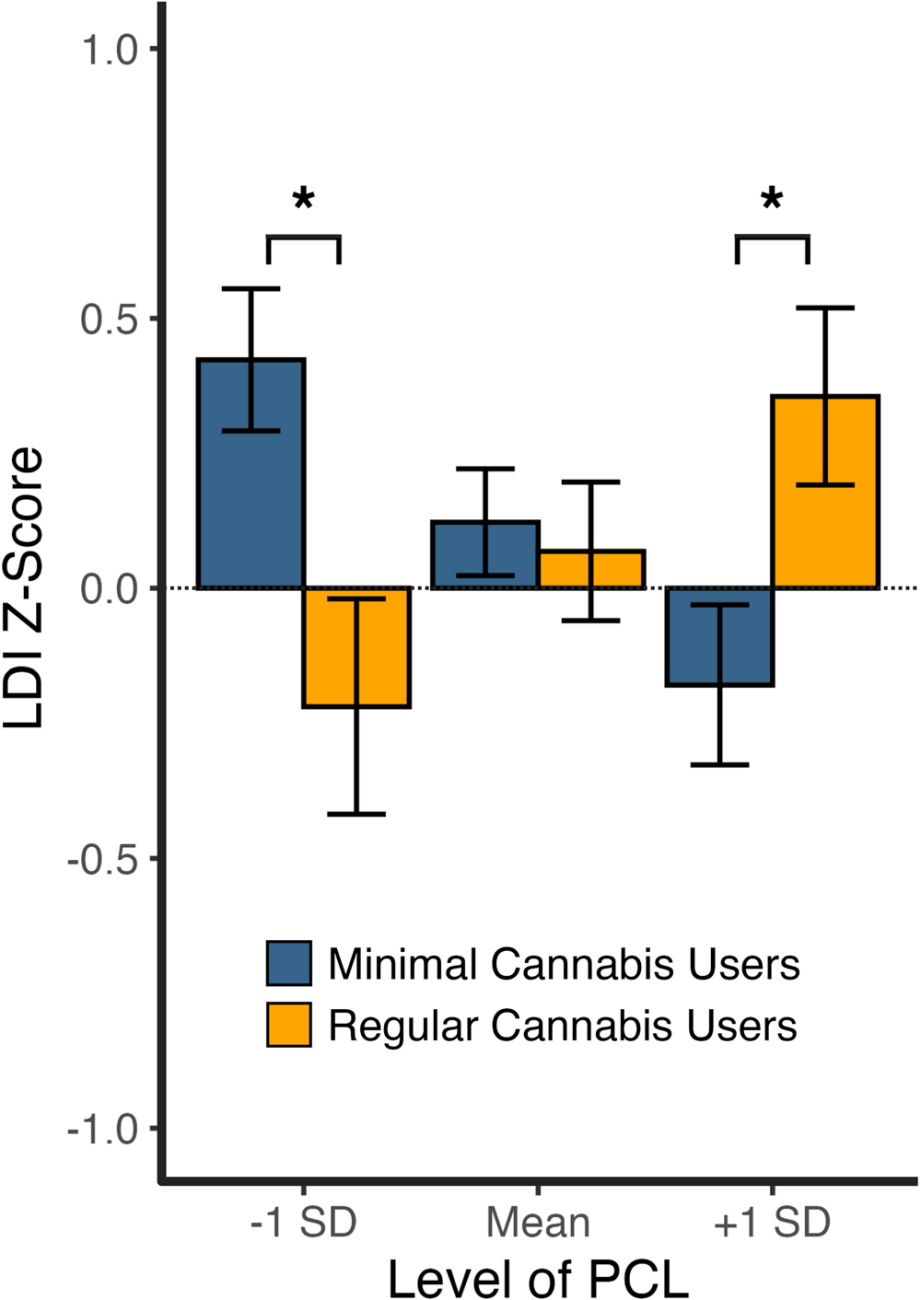
Mnemonic pattern separation performance comparing Minimal versus Regular Cannabis Users as a function of low PCL (-1 SD), Mean PCL, and high PCL (+ 1 SD). LDI Z-Score = Lure discrimination index Z-scored; which measures mnemonic pattern separation performance. Estimated marginal means are plotted for visualization purposes. LDI was Z-scored within sample cohort (see Method). PCL-5 = PTSD Symptom Checklist for DSM-5. *p < .05. Error bars reflect standard error. See online article for the color version of this figure. Figure was created using R (*ggplot2*) and BioRender.com.

To visualize the interaction between cannabis use and PTSD symptom severity on mnemonic pattern separation, we conducted a post-hoc analysis, comparing pattern separation ability between minimal and regular cannabis users across individuals with low (below 1 SD from the mean), mean (between -1 and +1 SD from the mean), and high (above 1 SD from the mean) PTSD symptoms. As shown in Fig. 3, at low PTSD symptoms (-1SD), pattern separation ability was significantly lower for regular cannabis users compared to minimal users (*t* = -2.69, *p* = .008). In contrast, at high PTSD symptoms (+ 1 SD), pattern separation ability was significantly higher for regular users than minimal users (*t* = 2.42, *p* = .017). At Mean levels of PCL, there were no group differences (*t* = -0.33, *p* = .74).

### Exploratory analyses: role of sex on moderating effect of PTSD on pattern separation

Due to the importance of elucidating possible sex differences, we next conducted an exploratory analysis with sex added as a factor in our model; the PTSD x Cannabis Use interaction remained significant, *F* (1, 102) = 14.37, *p* < 0.001, ${\eta }_{p}^{2}=.12$. We also observed a PTSD x Cannabis Use x Sex interaction which approached significance, *F* (1, 102) = 3.87, *p* = 0.053, ${\eta }_{p}^{2}=.037$. Post-hoc simple effects analyses indicate that the PTSD x Cannabis interaction effect was stronger in women: Increasing PTSD severity in women was associated with higher pattern separation in regular cannabis users (*p* = 0.005) but lower pattern separation in minimal-cannabis users (*p* = 0.031). In men, increasing PTSD severity was associated with lower pattern separation in minimal-cannabis users (*p* = 0.048), but PTSD severity had no relationship with pattern separation in regular cannabis users (*ps* = 0.62; see Supplementary Fig. 1). However, due to the relatively small number of women in the sample (*n* = 31), these results should be taken cautiously.

## Discussion

The aim of the current investigation was to test whether regular cannabis use would be associated with significant disruptions in hippocampal-dependent memory processes in individuals with elevated PTSD symptoms. We observed three primary results. First, we replicated the finding that individuals with elevated PTSD symptoms have impaired hippocampal-dependent function as measured by pattern separation ability^30^. Second, we found that the PTSD association with pattern separation performance is moderated by cannabis use. Individuals in the minimal cannabis use group showed the expected dysfunction in mnemonic pattern separation performance. In contrast, and unexpectedly, individuals in the regular cannabis use group exhibited better mnemonic separation ability with increasing levels of self-reported PTSD symptoms. Self-reported history of frequency, form, and %-tetrahydrocannabinol (THC) content was not associated with PTSD or depression symptoms, indicating that differences in patterns of use do not explain the paradoxical PTSD-related variation in pattern separation performance between these groups. Additionally, post-hoc analyses showed that individuals in the regular cannabis use group had significantly poorer pattern separation ability compared to the group with minimal cannabis use at lower levels of PTSD symptoms. Collectively, our findings highlight a complex relationship between PTSD symptoms, cannabis use, and hippocampus-dependent mnemonic pattern separation ability.

Regular cannabis use has been associated with various cognitive impairments^34,49,68^. Acute cannabis use in adolescence is associated with impairment in working memory and learning ability during adolescence^31,33^. Regular consumption of cannabis, particularly at high doses, has been linked to deficits in attention, memory, and executive functions in healthy individuals as well as a reduction in hippocampal volume^41,43,69,70^; although this negative impact is not always observed^71,72^. Some of these detrimental effects on cognitive performance and the hippocampus may be diminished after periods of abstinence or are less likely in users of cannabis products with higher cannabidiol (CBD) concentrations^73–76^. The effects of cannabis on cognition and brain structure likely depend on the age of use in addition to the amount, duration, frequency, and type (i.e., THC vs CBD ratio) of cannabis used^77,78^. Here, we show that regular cannabis use in those who are relatively asymptomatic from PTSD and depression symptoms have impaired mnemonic pattern separation ability. These findings are consistent with a double-blind, placebo-controlled within-subjects trial, which demonstrated that acute THC consumption immediately prior to the learning phase in a modified version of the MST impairs the encoding of perceptual details to discriminate between previous stimuli and semantically similar but different stimuli compared to placebo^79^.

Our finding of low levels of mnemonic pattern separation in minimal cannabis users with elevated PTSD are consistent with previous findings^30^ and with theoretical models that implicate impaired hippocampal functioning in the etiology and maintenance of PTSD^3,5^. PTSD symptom severity is associated with reduced hippocampal volume^80,81^, altered hippocampus activity and resting-state connectivity prospectively predict future PTSD symptoms following trauma^82,83^, and hippocampus structure and function are modified following treatment^84,85^. Hippocampal-dependent pattern separation impairment is a key mechanism underlying the overgeneralization of fear and contextual learning^6,86,87^. Overgeneralization of fear has been implicated in PTSD, wherein otherwise innocuous or “safe” stimuli are perceived as threatening, potentially leading to maladaptive avoidance behaviors which may strengthen the association of that stimuli with threat^88^. Our results are also consistent with prior imaging studies examining fear generalization in individuals with PTSD via overgeneralization gradients corresponding to the duration of altered fMRI BOLD signal change in regions employed in fear conditioning, including the hippocampus^89–92^.

The finding that regular cannabis use was associated with improved pattern separation ability in those reporting more severe PTSD symptoms was unexpected. One possible explanation for this observation is that the endocannabinoid system is altered by trauma exposure and in PTSD^93^. For example, meta-analytic evidence suggests that PTSD is associated with greater baseline anandamide (AEA) and 2-arachidonoglycerol (2-AG) endocannabinoid levels in serum and blunted release in response to stress^94,95^; while depression in some populations is associated with reduced serum levels of endocannabinoids^96,97^. Furthermore, positron emission tomography studies have reported increases in CB1R receptor availability in the brain in individuals with PTSD and depression patients relative to psychiatrically healthy individuals^98–100^. Moreover, animal studies consistently show that reduced endocannabinoid signaling at the CB1 receptor associated with emotional memory impairment can be normalized by increasing CB1 receptor signaling^101^. Thus, differences in baseline endocannabinoid levels and receptor availability suggest that patients with PTSD and depression symptoms may respond differently to cannabis use compared to non-symptomatic controls.

A second potential mechanism underlying the paradoxical PTSD-related improvement in pattern separation is the potential role of CBD in ameliorating cognitive impairment in neuropsychiatric disorders^102,103^. Experimental and meta-analytic evidence supports the hypothesis that CBD may reduce cognitive impairment associated with a range of different neuropsychiatric disease models^104,105^. For example, oral administration of CBD was associated with increased resting-state functional connectivity of the frontostriatal circuit^106^, improvement in working memory^107^, and enhancement of verbal memory^108^. A third possibility is dose-response effects of cannabis on cognition and memory. Rodent models suggest that hippocampal neurogenesis and the rescue of age-related deficits in memory is a result of biphasic dose-response to THC^109^. Consistent with the importance of dose in cannabis effects, recent work has shown regular and heavy cannabis use in healthy individuals is associated with impaired fear extinction^110^, but acute low dose THC appears to improve fear extinction and may help modulate amygdala reactivity in response to fear in those with PTSD^111,112^. In sum, existing work provides support for the notion that underlying differences in endocannabinoid signaling in PTSD may result in altered sensitivity or dose response shifts to CB1 agonists, resulting in differential responses to cannabis compared to psychiatrically healthy controls.

The paradoxical results observed in this study are in line with cannabis use associations in other neuropsychiatric disorders, suggesting transdiagnostic effects of cannabis on cognitive performance^113,114^. Regular and long-term cannabis use has been associated with improved, not impaired, cognitive performance in several neuropsychiatric disorders, including schizophrenia^115^, and bipolar disorder^116^. For example, individuals with Bipolar I disorder and comorbid Cannabis Use Disorder perform significantly better on several neurocognitive measures including attention, processing speed, and working memory^117^. Additionally, lifetime cannabis use is associated with better working memory and processing speed in individuals with Schizophrenia, even after controlling for the effect of antipsychotic medications^118^. Here, we report that pattern separation performance in regular cannabis users is also higher in individuals reporting elevated PTSD symptoms. We further observed that the positive association was not specific to PTSD symptoms by showing that this pattern was no longer significant after controlling for comorbid depressive symptoms. In our sample, PTSD and depression symptoms were highly collinear (*r* = 0.80), which is consistent with the high level of comorbidity between PTSD and depression^119,120^. Thus, we are unable to effectively separate PTSD from depression. However, our results support the hypothesis that the endocannabinoid system plays a role in cognition and highlight the need for future research to disentangle the distinct and overlapping contributions of various neuropsychiatric symptoms on the cognitive enhancements observed with regular cannabis use.

From a clinical perspective, isolating the path by which cannabis exerts potential negative and positive influences on cognition and what the implications are for clinical outcomes for PTSD will be important. Although we observed higher performance of hippocampal-dependent mnemonic pattern separation in regular cannabis users, this occurred only at higher levels of PTSD symptoms, suggesting that regular cannabis use is not improving self-reported psychiatric symptoms. Moreover, we conducted a cross-sectional study design, and therefore we cannot determine whether cannabis is a causal agent in producing improved cognition or modifies PTSD symptoms. Evidence is mixed regarding the beneficial impact of cannabis on PTSD. Some studies suggest a reduction in hyperarousal symptoms and significantly better PTSD symptom remission rates in regular users^121^. Whereas other studies show no long-term effect^122^, or a deleterious effect of cannabis on PTSD symptoms^123^. Yet, there remains a strong interest by patients and clinicians in the clinical application of cannabis to treat PTSD^124,125^. Despite a small number of individual studies indicating potential beneficial effects of cannabis in individuals with PTSD, meta-analyses highlight the lack of high-quality clinical trial results addressing the effects of cannabis on cognitive functioning and clinical outcomes in PTSD^126^. Currently, both the U.S. Department of Defense and U.S. Department of Veterans Affairs Clinical Practice Guidelines recommend against the use of cannabis in the treatment of PTSD due to the lack of evidence supporting efficacy^127^, underscoring the need to better understand the mechanisms by which cannabis may have salutary versus deleterious impacts on PTSD symptoms and cognition^128^.

Several limitations in the present study should be mentioned. First, we relied on self-report measures of cannabis use to define our groups, and urine test validation of THC was limited to the first cohort. Longitudinal investigations with experimental designs that validate %-THC/CBD concentrations, product label information, and quantification of cannabinoid metabolites in urine are clearly needed^129–131^. Second, our study included a small number of women, and therefore our exploratory finding of sex differences in pattern separation improvement within the regular cannabis use group should be interpreted cautiously. Evidence from animal and clinical studies suggests sex differences in the endocannabinoid system, including in PTSD populations^100,132–134^. Future research with larger samples of women and assaying reproductive hormone and cannabinoid levels is needed to clarify their combined impact on hippocampal-dependent mnemonic processes in PTSD^135^.

Collectively, our study contributes valuable insights into the intricate relationship between PTSD, cannabis use, and mnemonic pattern separation ability. The observed moderation effects of cannabis use on PTSD-associated cognitive dysfunction in cannabis users underscores the need for further research to elucidate the underlying mechanisms and potential therapeutic implications. Additionally, the differential impact of cannabis use in individuals with varying levels of PTSD symptoms highlights the complexity of these interactions, necessitating a comprehensive and nuanced approach to understanding the cognitive consequences of trauma and cannabis use.

## Supplementary information

Supplemental Materials

## Data Availability

Data will be made available upon reasonable request and with appropriate data use agreement.
