# Supplementary material for: Impaired mnemonic pattern separation associated with PTSD symptoms paradoxically improves with regular cannabis use
Source: Npj Ment Health Res. 2025 Apr 24;4:13. doi: 10.1038/s44184-025-00126-w (PMC12022266; doi:10.1038/s44184-025-00126-w)
Supplement: Supplementary file 1 — Supplemental Materials [file 44184_2025_126_MOESM1_ESM.pdf]

Supplementary Materials for Impaired Mnemonic Pattern Separation Associated with PTSD Symptoms Paradoxically Improves with Regular Cannabis Use

Jacob Ross[1,2]; Bruna Cuccurazzu[3]; Dylan Delmar[3], Christian Cortes[3] Giovanni Castillo[3]; Dean T. Acheson[3,4]; Dewleen G. Baker[3,4]; Victoria B. Risbrough[3,4]; Daniel M. Stout[3,4*]

[1] Department of Biological Sciences, University of California San Diego, San Diego, CA 92093 USA

[2] Research Service, San Diego ORD VISN 22, VA San Diego Healthcare System, San Diego, CA 92161 USA

[3] Department of Psychiatry, University of California San Diego, San Diego, CA 92093 USA

[4] Center of Excellence for Stress and Mental Health, VA San Diego Healthcare System, San Diego, CA 92161 USA

Supplementary Tables

Supplemental Table 1. General linear model in the larger (*n*=79) sample subset only

**Omnibus Model**			
	***F*-value**	***p*-value**	η_p^2
PTSD Severity	0.06	0.81	0.001
Cannabis Group	1.03	0.31	0.013
PTSD x Cannabis	11.32	0.001	0.130
**Simple Effects**			
	β	***t*-value**	***p*-value**
Minimal Cannabis Users	-0.343	-2.40	0.019
Regular Cannabis Users	0.396	2.37	0.020

Note. Analyses conducted on the larger sample subset recruited for this study (*n*=79 after removal of outliers and missing data (see Methods section in Main Report).

Supplemental Table 2. PCL-5 Reexperiencing x Cannabis Group General linear model

**Omnibus Model**			
	***F*-value**	***p*-value**	η_p^2
PCL-Re-experiencing	0.02	0.88	<0.001
Cannabis Group	0.10	0.76	0.001
PCL-Re-experiencing x Cannabis	13.63	<0.001	0.113
**Simple Effects of PCL-Re-experiencing**			
	β	***t*-value**	***p*-value**
Minimal Cannabis Users	-0.361	-3.11	0.002
Regular Cannabis Users	0.333	2.25	0.026

Note. PCL=PTSD Symptom Checklist.

Supplemental Table 3. PCL-5 Avoidance x Cannabis Group General linear model

**Omnibus Model**			
	***F*-value**	***p*-value**	η_p^2
PCL-Avoidance	0.10	0.76	0.001
Cannabis Group	0.15	0.70	0.001
PCL-Avoidance x Cannabis	12.57	<0.001	0.105
**Simple Effects of PCL-Avoidance**			
	β	***t*-value**	***p*-value**
Minimal Cannabis Users	-0.309	-2.66	0.009
Regular Cannabis Users	0.370	2.43	0.017

Note. PCL=PTSD Symptom Checklist.

SUPPLEMENTARY TABLES CONTINUED

Supplemental Table 4. PCL-5 Negative Cognition and Mood x Cannabis Group General linear model

**Omnibus Model**			
	***F*-value**	***p*-value**	η_p^2
PCL-Negative Cognition & Mood	0.07	0.79	0.001
Cannabis Group	0.11	0.74	0.001
PCL-Negative Cog/Mood x Cannabis	11.38	0.001	0.096
**Simple Effects of PCL-Negative Cognition & Mood**			
	β	***t*-value**	***p*-value**
Minimal Cannabis Users	-0.348	-2.87	0.005
Regular Cannabis Users	0.297	2.01	0.047

Note. PCL=PTSD Symptom Checklist.

Supplemental Table 5. PCL-5 Hyperarousal x Cannabis Group General linear model

**Omnibus Model**			
	***F*-value**	***p*-value**	η_p^2
PCL-Hyperarousal	0.01	0.92	<0.001
Cannabis Group	0.07	0.79	0.001
PCL- Hyperarousal x Cannabis	8.08	0.005	0.070
**Simple Effects of PCL-Hyperarousal**			
	β	***t*-value**	***p*-value**
Minimal Cannabis Users	-0.291	-2.56	0.012
Regular Cannabis Users	0.271	1.68	0.097

Note. PCL=PTSD Symptom Checklist.

SUPPLEMENTARY FIGURES

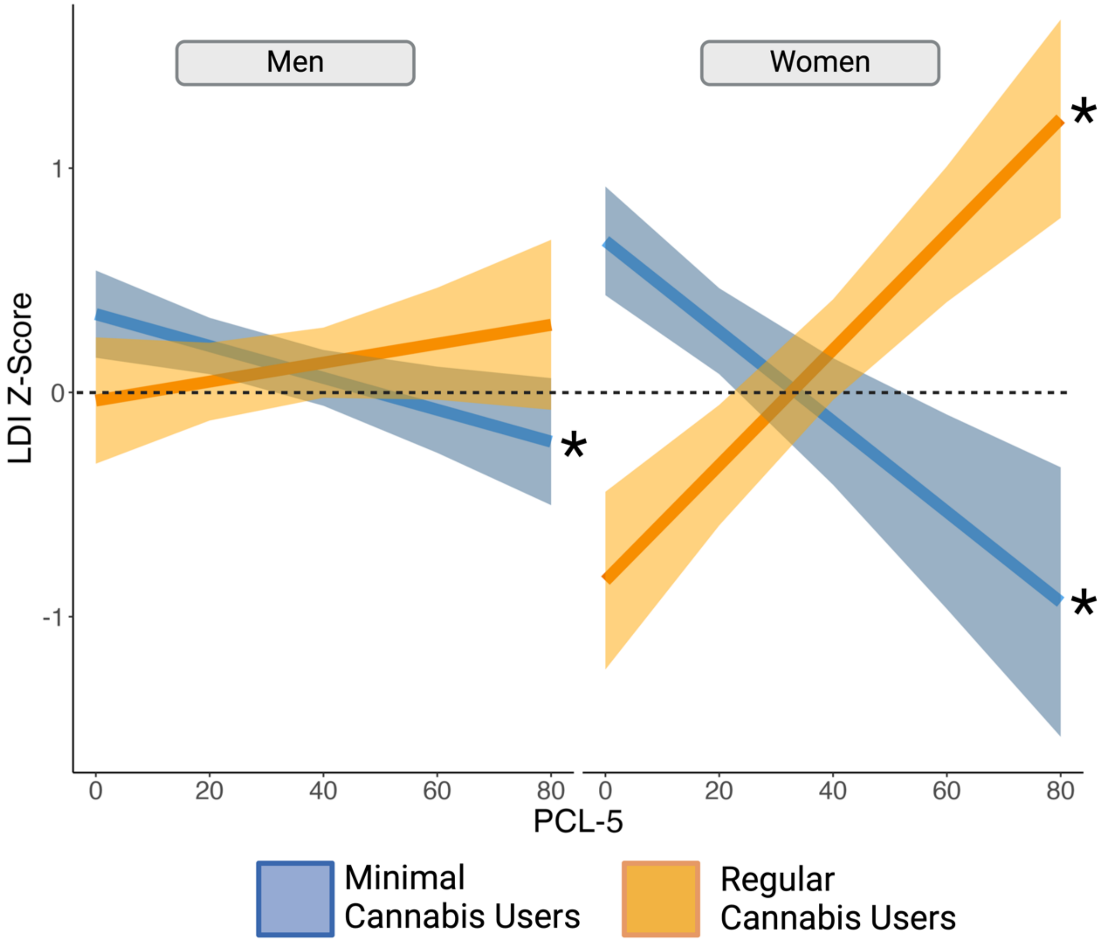

Supplemental Figure 1.

Mnemonic pattern separation performance as a function of PTSD symptom severity and Sex in individuals who minimally use cannabis and in individuals with regular cannabis use. *Note*. LDI Z-Score = Lure discrimination index Z-scored; which measures mnemonic pattern separation performance. LDI was Z-scored within sample cohort (see Method). PCL-5 = PTSD Symptom Checklist for DSM-5. * Simple slope effects: $p < .05$. Ribbon reflects standard error of the mean. See online article for the color version of this figure. Figure was created using R (*ggplot2*) and BioRender.com.
